# Immune heterogeneity of head and tail pancreatic lymph nodes in non-obese diabetic mice

**DOI:** 10.1038/s41598-019-45899-1

**Published:** 2019-07-05

**Authors:** Xiaofei Li, Asher Bean, Mayuko Uehara, Naima Banouni, Moufida Ben Nasr, Vivek Kasinath, Liwei Jiang, Paolo Fiorina, Reza Abdi

**Affiliations:** 1 Transplantation Research Center, Renal Division, Brigham and Women’s Hospital, Harvard Medical School, Boston, MA 02115 USA; 2 Key Laboratory of Combinatorial Biosynthesis and Drug Discovery, Ministry of Education, and Wuhan University School of Pharmaceutical Sciences, Wuhan, 430071 China; 3 Nephrology Division, Boston Children’s Hospital, Harvard Medical School, Boston, MA USA

**Keywords:** Immunology, Molecular biology

## Abstract

The pancreatic lymph node is critical to the pathogenesis of autoimmune diabetes, as it constitutes the initial site for the priming of autoreactive T cells. In this study, we compared the histopathology of the head pancreatic lymph node (HPLN) to the tail pancreatic lymph node (TPLN) in NOD mice. HPLNs and TPLNs were harvested from 4 week-, 8 week-, and 12 week-old NOD mice, and their microvasculature, extracellular matrix, and immune cell subsets were characterized. The percentages of B cells and antigen-presenting cells (APCs) were much higher in the HPLN, as compared to the TPLN. Notably, the HPLNs of 12 week-old mice were characterized by greater expansion of high endothelial venules (HEVs) and lymphatic vessels in comparison to the TPLNs. Finally, we observed a higher density of extracellular matrix (ECM) fibers surrounding the lymphatic vasculature in the HPLNs than in the TPLNs. These data for the first time demonstrate that the HPLN possesses a different immune microanatomy and organization from the TPLN. These novel observations unveil a major phenotypic difference between two types of LNs from the same organ and may highlight an independent fundamental role played by each PLN during the establishment of T1D.

## Introduction

The presentation of islet antigens by APCs to T cells is central to the pathogenesis of autoimmune diabetes in the draining pancreatic lymph nodes (PLN), resulting in the activation of autoreactive T cells^1,2^. Accordingly, several studies indicate the critical role of the PLN in the pathogenesis of autoimmune diabetes as it constitutes the initial site of priming of diabetogenic-autoreactive T cells^1,3–9^. As of to date, LN of any given organ is treated equally including the PLN. LN are connected to organs via afferent lymphatic ducts facilitating the initiation of adaptive immune responses upon their encounter with dendritic cells-associated antigens. The anatomy of LNs is extremely specialized organs orchestrating a well-coordinated immune cell trafficking and homeostasis. T cells home to the LNs via high endothelial venules (HEVs) on a daily basis. HEVs express a series of glycoproteins known as peripheral node addressin (PNAd), which engages with L selectin expressed on the surface of T cells, an interaction that allows the T cells to enter the LN. Naïve T cells that have entered the LN may interact with APCs, which may carry autoantigens (as in the case of T1D). The integrity of the HEV is monitored by surrounding stromal cells known as fibroblastic reticular cells, or FRCs^10^. On the other hand, LNs are monitoring the milieu of the tissue continuously by surveying molecules that enter and pass through the subcortical sinus (SCS) of the LN via afferent lymphatic ducts. The SCS contains a large number of CD169^+^ macrophages^11,12^, known for their efficient ability to internalize antigens within the lymph^11^. These macrophages can present antigens to B cells, dendritic cells, or T cells in the lymph node to promote the immune response^11,13^. We demonstrated for the first time the existence of substantial immunohistological differences between the head and the tail of PLN of NOD mice which may unveil their distinct role during the pathogenesis of T1D. These data also set forth a concept that different LNs that drain a given organ may be characterized by separate ontogeny and their roles may differ in various diseases, including diabetes and cancer. Major initiatives for the procurement of organs, including the PLN, from T1D patients have been initiated to gain greater insight into the role of pathogenic human T cells. However, assuming that every PLN possess similar characteristics may confound the results.

## Research Design and Methods

### Mice

NOD/ShiLtJ (NOD) at 4, 8 weeks and 12 of age, were purchased from the Jackson Laboratory (Bar Harbor, Maine). All mice were cared for and used in accordance with the institutional guidelines and regulations approved by the Institutional Animal Care and Use Committee of Brigham and Women’s Hospital, Harvard University, Boston, MA (protocol number: 2016N000167).

### Immunofluorescence staining

PLNs were embedded in optimum cutting temperature compound and stored at −80 °C. Then, the frozen sections were cut at 8μm and washed with PBS for 5 min. Samples were blocked with 3% (vol/vol) bovine serum albumin in PBS and incubated with primary antibodies overnight. Sections were washed 3 times with PBS and incubated with secondary conjugated antibodies at room temperature. The following antibodies were used for the staining: goat anti-PDPN (R&D Systems, 1:200), rat anti-Meca79 (Novus Biologicals, 1:200), rabbit anti-Fibronectin (Abcam, 1:300), rat anti-ERTR7 (Santa Cruz Biotechnology, 1:100), rabbit anti-collagen IV (Abcam,1:300), rat anti-B220 (Invitrogen, 1:200), rabbit anti-CD3 (Abcam, 1:250), rabbit anti-lyve-1 (Abcam, 1:300). Secondary antibodies were either FITC- or Cy3-conjugated (Jackson ImmunoResearch, 1:200). DAPI (VECTASHILED, Vector Laboratories) mixed in Prolong-Gold mounting media was used as a nuclear counterstain. Images were obtained by EvosFL Auto2 microscopy. All images were automatically processed using ImageJ (NIH) and split into RGB channels. Auto threshold was used to convert intensity values of the immunofluorescent staining into numeric data.

### Flow cytometry analysis

In order to characterize which kind of leukocytes are infiltrating the PLN, we collected the PLNs from 4 weeks, 8 weeks and 12 weeks old NOD mice and incubated them with 5 mL dissociating solution (1 mg/ml Collagenase D, 0.1 mg/ml DNase1). The single cell suspension was centrifuged at 1500 rpm for 5 minutes and resuspended in PBS at 1 × 10^7^ cells/ml for FACS staining. The following Abs were used: PE-Cy7-conjugated anti-CD11c, PE-Cy7-conjugated anti-CD11b, FITC-conjugated anti-lyve-1. Live and dead cells were discriminated using Pacific Blue-conjugated viability dye. All of the antibodies were purchased from BD (Becton Dickinson Franklin Lakes, NJ). Finally, the samples were acquired by FACS Canto II (BD Biosciences, Franklin Lakes, NJ) flow cytometer and data were analyzed using FlowJo V10.0 software.

### Statistics analysis

Two-way ANOVA and unpaired two-tailed t test were used for comparison of experimental groups. Differences considered to be significant when *p* < 0.05 (*p < 0.05, **p < 0.01). Prism software was used for data analysis and to prepare graphs (GraphPad). Data represent mean ± SD. Image J software was used for semi-quantitative analysis of immunofluorescent staining.

## Results

### Immunophenotypic characterization of HPLNs and LPLNs

**Figure 1:**
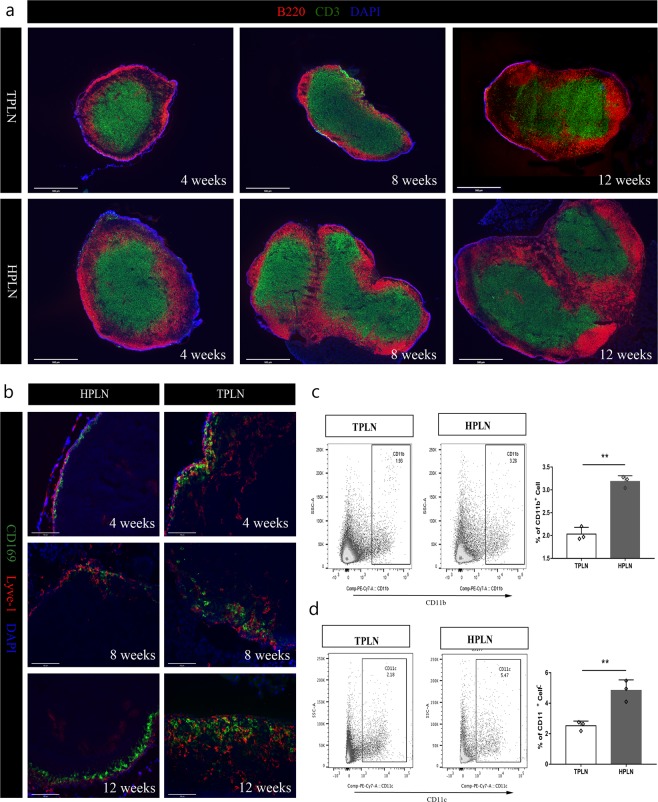
Characterization of immune cells in the TPLN and HPLN of NOD mice. (**a**) Co-immunostaining of B220 (red) and CD3 (green) in whole region of sectioned PLNs. Histology magnification was 4x, scale bars represent 500 µm. Images are representative of three independent experiments (n = 3). (**b**) TPLN and HPLN sections from respectively 4, 8 and 12 wks old NOD mice were co-immunostained for LYVE-1 (red) and CD169^+^ subcapsular sinus macrophages (green). Histology magnification was 20X and scale bars represent 100 µm. Image are representative of three independent experiments (n = 3). (**c**,**d**) Representative flow cytometric analysis and quantitative bar graphs of the expression of CD11b^+^ macrophages and CD11c^+^ dendritic cells in the PLNs of 12 weeks old NOD mice. All data are expressed as mean ± SD, *p < 0.05, **p < 0.01.

Our immunofluorescence analysis revealed the enlargement the HPLN as compared to TPLN at 8 and 12 weeks old NOD mice associated with an expansion of B and T cells in the HPLN (Fig. 1a). This was mainly characterized by an expansion of the B cell zone into the paracortical region (Fig. 1a and Supplementary Fig. 1b). Similar trends were observed in CD3^+^ T cells area in the HPLN, however, it did not reach statistical significance until 12 weeks (Supplementary Fig. 1c). Staining the PLNs for CD169^+^ (resident macrophages in the SCS), showed a marked increase in the HPLN of 12 weeks old NOD as compared to their counterparts TPLN (Fig. 1b, Supplementary Fig. 1d). There was also a trend in 4 and 8 weeks NOD mice as well. We further observed a significantly increased expression of CD11b^+^ cells within the HPLN of 12 weeks old NOD mice as compared to that of the TPLN (3.2 ± 0.1% within HPLN vs 2.0 ± 0.1% within TPLN, Fig. 1c, p < 0.01). Increased frequency of CD11c^+^ cells was also noted within the HPLN of 12 weeks old NOD mice (2.3 ± 0.2% vs 4.8 ± 0.4% in TPLN and HPLN, respectively, p < 0.01, Fig. 1d).

### Higher expansion of lymphatics and HEV in HPLN vs. TPLN

**Figure 2:**
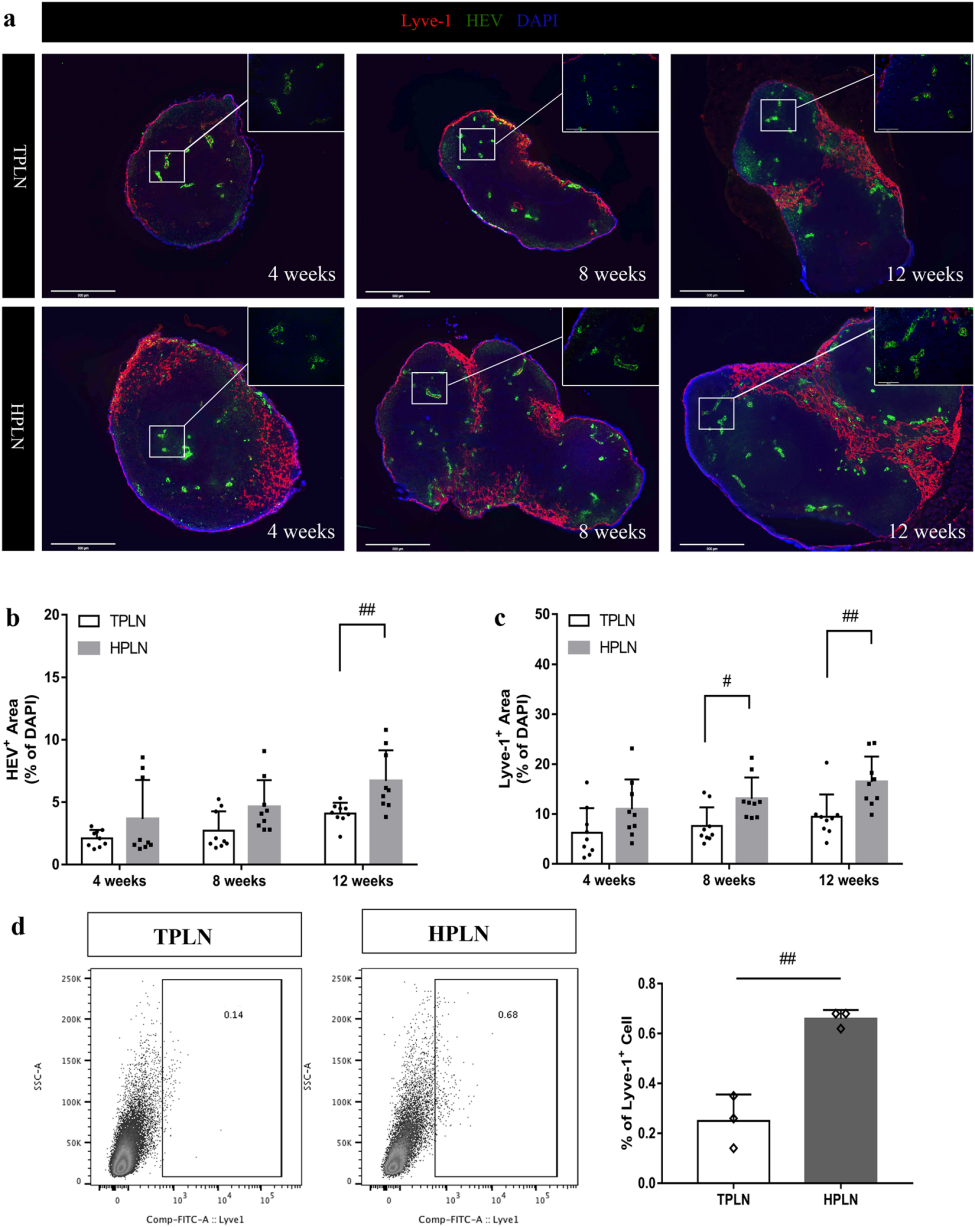
Comparative analysis of the lymphatic vessels and HEVs of HPLN and TPLN. (**a**) HPLN and TPLN sections from respectively 4, 8 and 12 wks old mice were co-stained with LYVE-1 (red) and HEV (green). Histology magnification 4× and 20×, scale bars represent 500 µm and 100 µm. Images are representative of three independent experiments (n = 3). (**b**,**c**) quantitative bar graphs of Lyve-1^+^ staining and Meca^+^ in HPLN vs TPLN, the quantification were performed by ImageJ, data are representative of n = 3 images per group and 3 sections per each LN were analyzed. (**d**) Representative flow cytometric analysis and quantitative bar graph showing the expression of Lyve-1^+^ cells in the PLNs of 12 weeks old NOD mice. All data are expressed as mean ± SD, *p < 0.05, **p < 0.01.

An expansion of HEVs within the HPLN was obsearved as compared to theri couterparts (Fig. 2a,b). The lymphatic vasculature also showed marked expansion in the HPLN as compared to their TPLN at 8 and 12 weeks in NOD mice (Fig. 2a,c). Similar trends were observed in the HPLN of 4 weeks old NOD as compared to their TPLN counterparts, although not statistically significant. The further flow cytometry analysis experiment confirmed an increase in the percentage of Lyve-1^+^ lymphatic endothelial cells at 12 weeks Fig. 2d).

### Lymphatic region of HPLN is highly enriched of podoplanin (PDPN) and ECM

**Figure 3:**
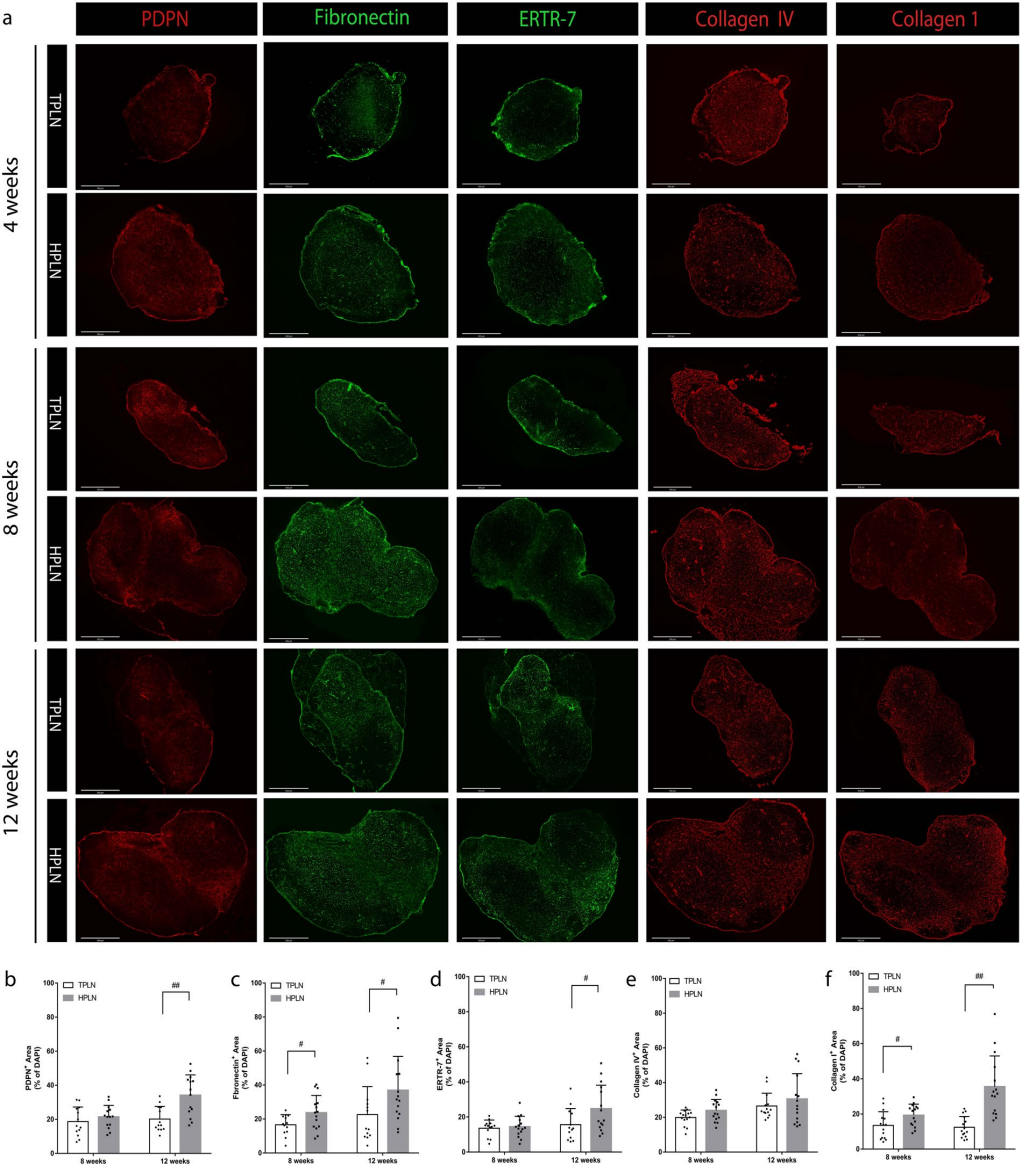
Comparative analysis of FRCs and the ECM organization between TPLN and HPLN. (**a**) Whole scan images of representative sections of TPLN and HPLN from respectively 4, 8 and 12wks old NOD mice showing the staining for PDPN (red), Fibronectin (green) and Collagen IV (red). Histology magnification 4x and Scale bars represent 500 µm. Images are representative of three independent experiments (n = 3). (**b–f**) Quantitative bar graphs of PDPN^+^, fibronectin^+^, ERTR-7^+^, collagen IV^+^ and collagen I^+^ staining in HPLN vs TPLN, the quantification positive areas were performed by ImageJ, data are representative of n = 5 images per group and 3 mice per group were analyzed. All data are expressed as mean ± SD, *p < 0.05, **p < 0.01.

FRCs are PDPN^+^ resident stromal cells that maintain the overall structure of the PLN by producing ECM. We stained the PLN sections for PDPN and various elements of ECM. While, there was no significant difference in terms of the size of PDPN^+^ resident stromal cells or ECM at 4 weeks (Fig. 3a), however, at 8 weeks we noted an increase in the expression of fibronectin and collagen IV in HPLN as compared to TPLN. At 12 weeks, we could observe significant increase in the PDPN^+^ cells (within the interstitium non-lymphatic area) as well as a marked increase in the expression of collagen IV, fibronectin and ERTR-7 staining within the two PLN structures (Fig. 3a–f, respectively). PDPN also marks lymphatics as well. The maximum increase in the expression of these markers was noted in the lymphatic area demonstrated by PDPN^+^ and Lyv1^+^ area (Supplementary Fig. 3a). Similar trends were observed in Collagen IV^+^ and ERTR-7^+^ area distribution (Supplementary Fig. 3b,c).

## Discussion

It is well established that the commencement of the autoimmune cascade occurs within the PLN which is particularly endowed with such specific organization triggering a rapid and effective immune response. Our main observation is that the PLN of NOD mice can be anatomically subdivided in two phenotypically different structures namely HPLN and TPLN with the former being more expanded and represent physiologically a more prominent environment along the progression of the autoimmune response. To this end, we observed that the HPLN are more enriched in B220^+^ cells and CD3^+^ cells than the TPLN, particularly a marked enlargement of the B cell zone into the paracortical region of the HPLN was observed at an advanced stage of the disease. Notably, a pronounced presence of macrophages and DCs, mainly higher populations of CD169^+^, CD11b^+^ and CD11c^+^ cells, were found within the HPLN rather than the TPLN and at later stage of the disease, which may emphasize the central role of HPLN during the autoimmune response. In fact, the major role of B cells and APCs in antigen presentation during the autoimmune response has been largely described where the PLN represent the central feature of a developmentally regulated presentation of islet beta cell antigen leading to the stimulation of islet reactive T cells^14–16^. These data suggest that evidence supporting the idea that the HPLN undergoes a greater degree of immune activation than the TPLN, we hypothesize that HPLN may represent the central feature of the whole PLN that plays a major role in promoting antigen presentation during the initiation of the autoimmune response. Another important observation resides within the distinctive morphology and the different organization of the lymphatic system within the 2 different features of the PLN (HPLN and TPLN) and during the progression of T1D. Characterizing the two types of vasculature important to the generation of immunity within the PLN, namely HEV and lymphatic structures within the 2 different features of PLN revealed that the HPLN contained larger areas occupied by HEVs and lymphatic vessels. Since HEVs play a critical role in facilitating the transfer of lymphocytes into the lymph node from the blood, the increased quantity of HEVs in the HPLN may allow for more the entrance of more lymphocytes into this specific lymph node and ultimately more antigen presentation^13^. Additionally, lymphatic vessels transport lymph fluid, including antigens, lymphocytes, and APCs, which are all known to participate in the activation of the immune response^17^. The more expanded lymphatic vasculature in the HPLN versus the TPLN also suggests a higher level of immune activity as well, as this permits more trafficking of antigens to the PLN. Notably, though both HEVs and lymphatic vasculature are observed in both the TPLN and HPLN, they seem to be significantly more concentrated within the HPLN and at an advanced stage of the autoimmune response. We Altogether, we herein demonstrated that the HPLN rather than the TPLN undergoes substantial reshaping of its immune microenvironment as well as its proper lymphatic system to further sustain the autoimmune response. Next, we noticed that as known as a specific marker for fibroblastic reticular cells (FRCs) in lymph nodes, the populations of PDPN^+^ cell in HPLN increased compared to the TPLN. Fibronectin, collagen IV and ERTR-7, are basement membrane extracellular matrix (ECM) components present within the FRC that showed consistent tendency with PDPN. In addition, increased expression of PDPN and ECM components were seen in the vicinity of the lymphatic vessels in the HPLN at an advanced stage of the onset of T1D (in 12 weeks old NOD). It has been acknowledged that lymphocytes and APC use the FRCs network as the route for trafficking within the node^18,19^. This phenomenon resembles our findings with respect to B and T cells, macrophages, HEVs, and lymphatic vasculature in the HPLN during the progression of the autoimmune response. Therefore, one can deduced from our data that the HPLN rather than the TPLN played critical role through an evolving and a drastic reshape of the lymphatic organization as well as of the adaptive immune composition facilitating thus the establishment of an appropriate immune response. These major observations led us hypothesize that the HPLN might represent a main or preferable site of the autoimmunity rather than the TPLN^20^. Furthermore, tissue fibrosis is the end result of excess ECM deposition induced by a variety of stimuli including autoimmune reactions, allergic responses and tissue injury^21^. There are different lymph nodes responsible for the drainage of the head, neck, body, and tail of the human pancreas which showed clinical relevance with pancreatic pathologies^22,23^. This is the first time we distinguished two different kinds of draining lymph nodes for the pancreas of NOD mice. Evaluating the status of these two types of lymph node may impact significantly the investigation of the course of autoimmunity during diabetes. The patchiness of infiltrates in the pancreas of T1D could be related to the differential role of these two types of LNs, as they drain separate specific areas of the pancreas. Our future studies will focus on determining if these preclinical data are clinically relevant. We will explore in depth whether our data might exert comparable significance with respect to examining the PLNs from a human T1D individual. If so, these data have major implications not only with respect to the pathogenesis of T1D, they may suggest that data gleaned from the analysis of T cells isolated from a mixed cell suspension from these two types of LN may be confounding. Further investigation of the functional contribution of each LN in generating pathological autoreactive T cells requires a significant amount of additional work. Another important ramification from these data is that the immune environment of the draining LN also has a major implication in the pathogenesis of other diseases, including cancer. Therefore, further identification of the differences in immune responsiveness between LNs that drain the same organ, and the implications that these findings may have on the pathogenesis of various diseases, may be extremely valuable.

## Supplementary information

Supplemental Figure 1, 2, 3

## Data Availability

All data generated or analyzed during this study are available from the author on reasonable request.
