# Supplementary material for: Immune heterogeneity of head and tail pancreatic lymph nodes in non-obese diabetic mice
Source: Sci Rep. 2019 Jul 5;9:9778. doi: 10.1038/s41598-019-45899-1 (PMC6611787; doi:10.1038/s41598-019-45899-1)
Supplement: Supplementary file 1 — Supplemental Figure 1, 2, 3 [file 41598_2019_45899_MOESM1_ESM.docx]

**Title:** Immune heterogeneity of head and tail pancreatic lymph nodes in non-obese diabetic mice

**Authors**: Xiaofei Li^1,2^，Asher Bean^1^，Mayuko Uehara^1^，Naima Banouni^1^，Moufida Ben Nasr^1,3^, Vivek Kasinath^1^, Liwei Jiang^1^, Paolo Fiorina^1,3^, Reza Abdi^1*^

**Affiliations**: ^1^Transplantation Research Center, Renal Division, Brigham and Women’s Hospital, Harvard Medical School, Boston, MA 02115, USA; ^2^Key Laboratory of Combinatorial Biosynthesis and Drug Discovery, Ministry of Education, and Wuhan University School of Pharmaceutical Sciences, Wuhan, 430071, China; ^*^Corresponding author; ^3^Nephrology Division, Boston Children's Hospital, Harvard Medical School, Boston, MA, USA

**Address for correspondence:**

Reza Abdi, MD

Transplantation Research Center, Brigham and Women’s Hospital

221 Longwood Ave, Boston MA 02116, USA

Tel: 617-732-5259,

Fax: 617-732-5254,

Email: [rabdi@rics.bwh.harvard.edu](mailto:rabdi@rics.bwh.harvard.edu)

Supplemental Figure 1


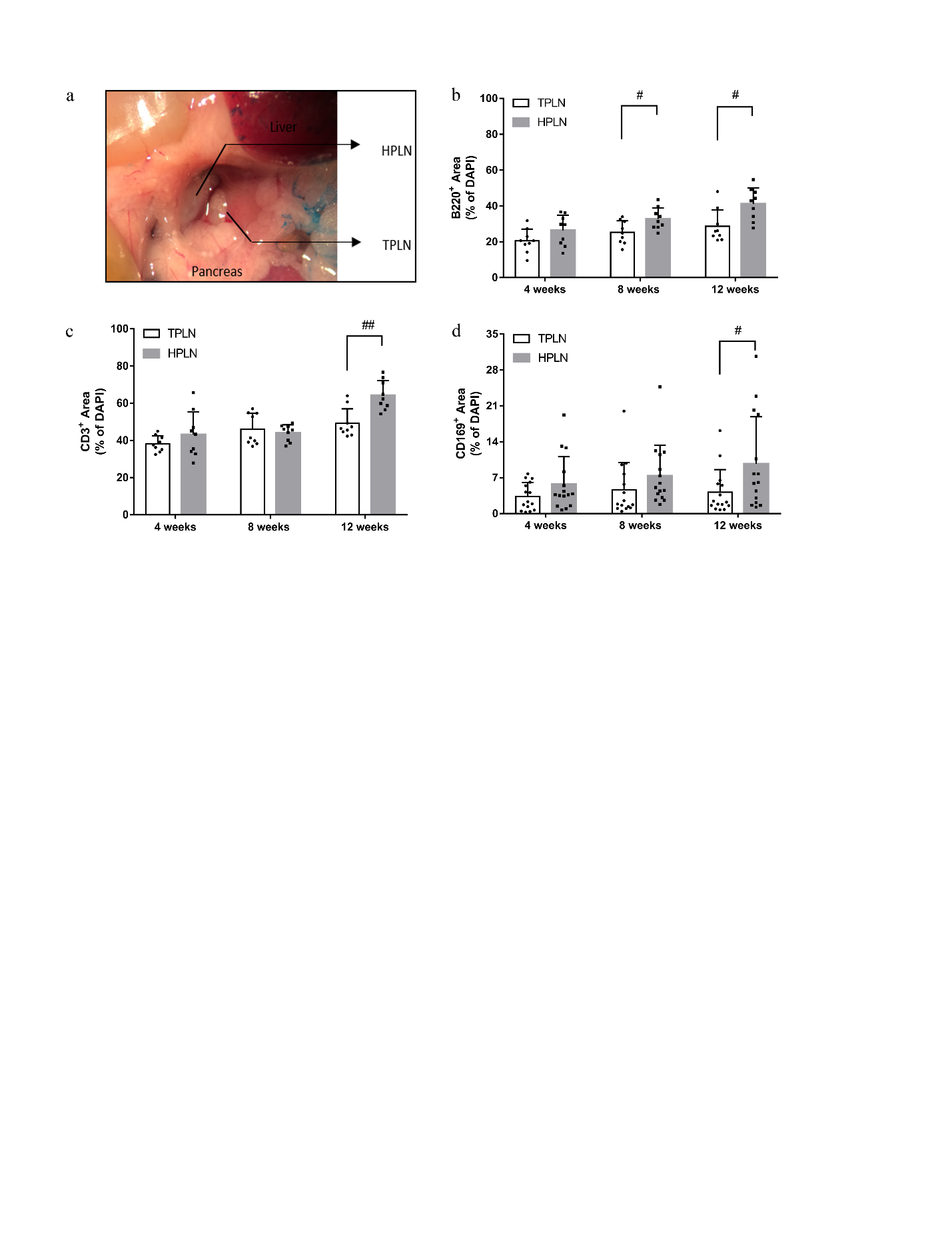


**Fig.1 Characterization and quantification of T cells and B cells of HPLN and TPLN in vivo.**

(a) Surgical schematic showing the anatomical positions of HPLN, TPLN and pancreas in a mouse. (b-d) Quantification of the percentage of B220^+^ area, CD3^+^ area and CD169^+^ area in comparison to DAPI nuclear staining in HPLN vs TPLN using ImageJ software (n=3, 3 sections per each LN per mouse, mean ± SD, student t-test, *p < 0.05).

Supplemental Figure 2


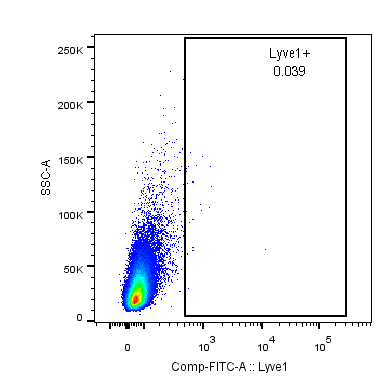


**Fig.2 Flow Cytometry Plot of Isotype Control for Lyve-1 Antibody**

Supplemental Figure 3


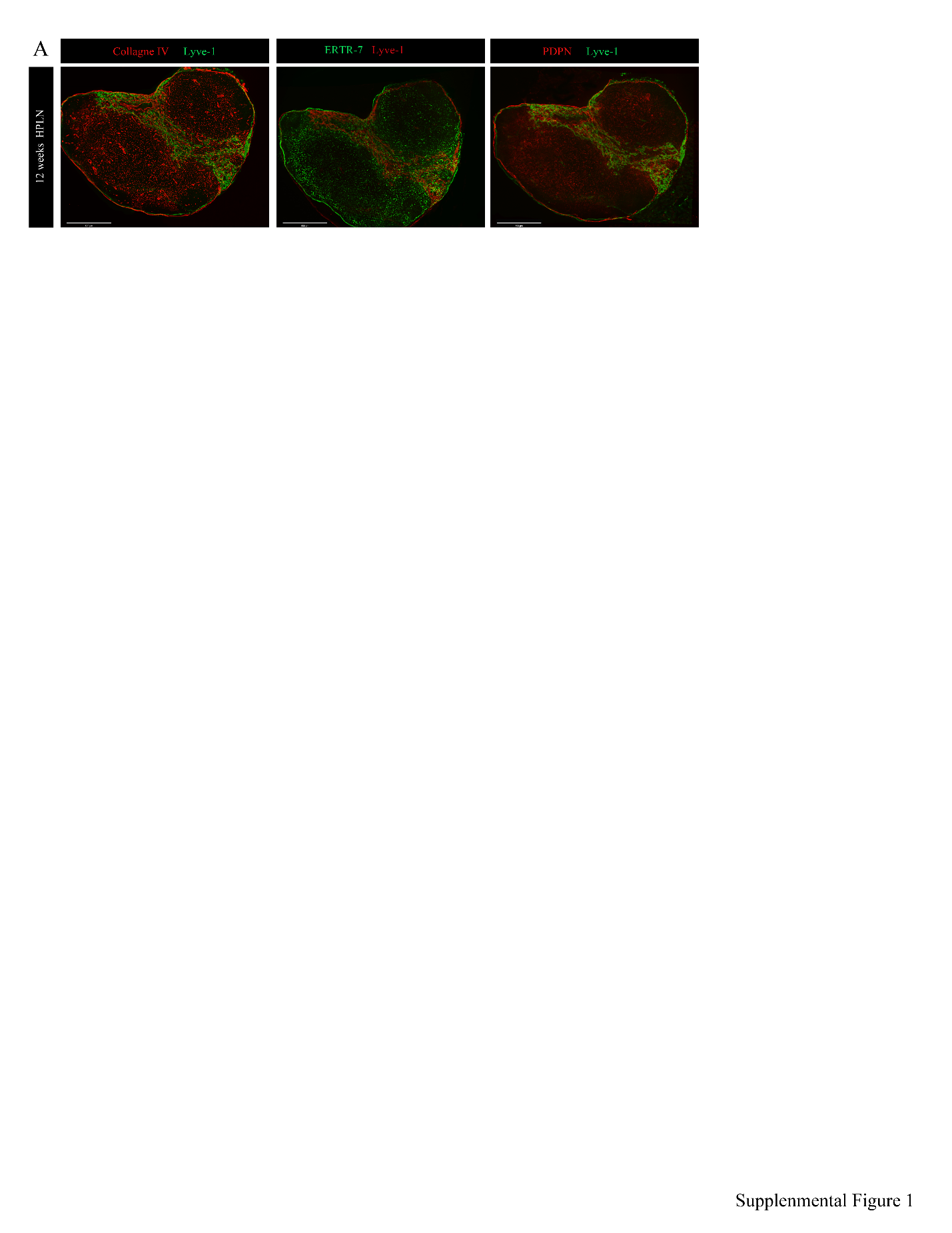


**Fig.3 ECM deposited around the lymphatic area.** Co-immunostaining of (a) Collagen IV (red) and Lyve-1 (green), (b) ERTR-7 (green) and Lyve-1 (green) and (c) PDPN (red) and Lyve-1 (Green) in whole region of sectioned PLNs**.**
